# Primary isolated extramedullary plasmacytoma of colon

**DOI:** 10.1186/1477-7819-5-47

**Published:** 2007-04-30

**Authors:** Vishal Gupta, Brindaban Nahak, Puja Sakhuja, Anil K Agarwal, Nirmal Kumar, Pramod K Mishra

**Affiliations:** 1 Department of Gastrointestinal Surgery, Gobind Ballabh Pant Hospital and Maulana Azad Medical College, New Delhi, India; 2 Department of Pathology, Gobind Ballabh Pant Hospital and Maulana Azad Medical College, New Delhi, India; 3 Department of Gastroenterology, Gobind Ballabh Pant Hospital and Maulana Azad Medical College, New Delhi, India

## Abstract

**Background:**

Extramedullary plasmacytoma is an uncommon entity that most commonly involves nasopharynx or upper respiratory tract. Involvement of the gastrointestinal tract occurs in approximated 10% of cases. Isolated primary plasmacytoma of colon is rare and only 7 well documented cases have been reported in the literature.

**Case presentation:**

We present a case of 42 year male who presented with diarrhea. Multiple colonic strictures were found on investigation. Colonoscopic biopsy was not helpful in making any specific diagnosis. Patient underwent subtotal colectomy. Isolated primary colonic plasmacytoma was found on histopathological examination.

**Conclusion:**

Plasmacytoma is known to occur in extra osseous sites. Primary colonic plasmacytoma, however, is a rare clinical entity. Primary colonic plasmacytoma may have varying clinical presentations including multiple colonic strictures that may mimic colonic tuberculosis or inflammatory bowel disease. Although these cases are rare, treating physician as well as radiologist, and pathologist should be aware of this entity.

## Background

According to World Heath Organization [1] plasma cell tumors have been classified in to two main groups: multiple myeloma and plasmacytoma. Plasmacytoma includes solitary plasmacytoma of bone and solitary extramedullary plasmacytoma.

Solitary extramedullary plasmacytoma represents a rare disease and diagnostic criteria and natural history are not very well defined at present. They are most commonly found in the oral cavity and upper respiratory tract [2]. **A**bout 10% of these tumors have been reported in gastrointestinal tract [3,4]. Involvement of colon and rectum is extremely rare and so far only 20 cases of extramedullary plasmacytoma of colon and rectum have been reported in the literature. However, only 7 of these cases have adequate clinical and other details.

## Case presentation

A 42 year old male presented in July 2004 with 8 months history of diarrhea, progressive weight loss and malaise. Diarrhea was intermittent, watery with a frequency of 3–4 times/day, without fresh or altered blood. It was associated with crampy lower abdominal pain. There was history of weight loss of 5 kg in last 8 months. There was no history of anorexia, fever, or alternating constipation. He had received anti tubercular treatment elsewhere thrice (1979 – 1 year, 1992 – 1 year, 2002 – 1 year) for suspected intestinal tuberculosis without any confirmatory evidence. Patient did not have any details about his past illness or treatment.

General physical examination was normal except for pallor. Per rectal examination including proctoscopy was normal. Hemoglobin was 6 gram per dl. Other laboratory investigations including chest X-ray, were with in normal limits.

**Figure 1:**
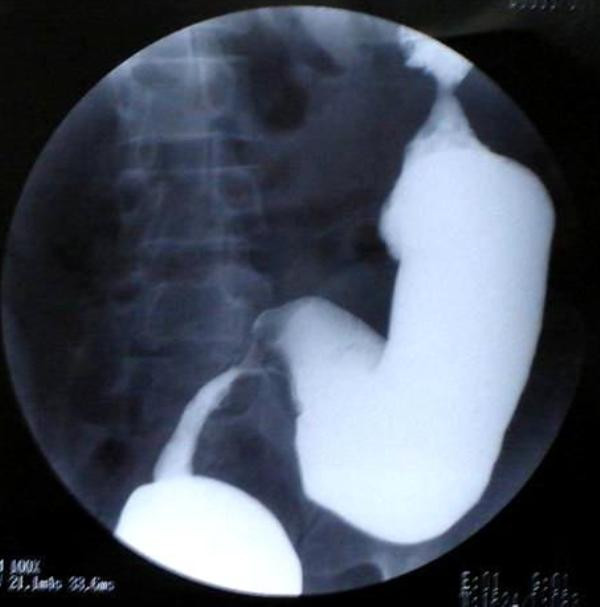
Stricture involving rectosigmoid area.

**Figure 2:**
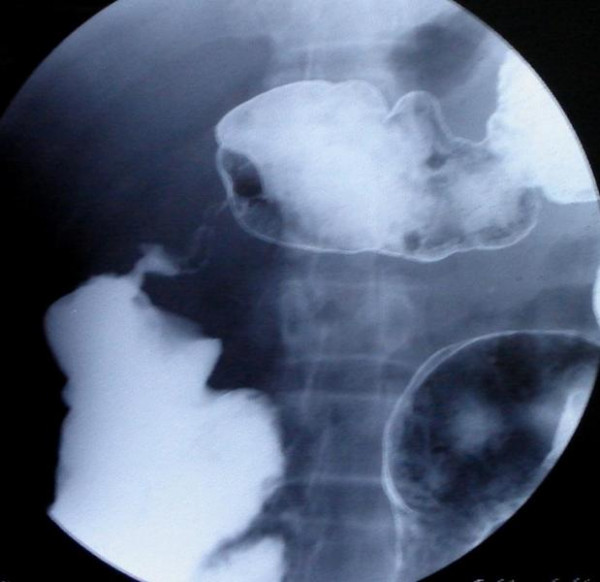
Stricture proximal transverse colon.

Patient underwent Barium enema examination in 2002 that showed multiple strictures in colon. As patient did not have films, this test was repeated and again it showed similar findings (Fig 1, 2). There were three strictures in colon involving sigmoid colon, ascending colon, and proximal transverse colon. Barium meal follow through was done to rule out small intestinal involvement and was normal.

A non passable stricture with ulcerated overlying mucosa at 18 cm of the anal verge was revealed with colonoscopy, whereas the rest of the visualized part of rectum and colon was normal.

**Figure 3:**
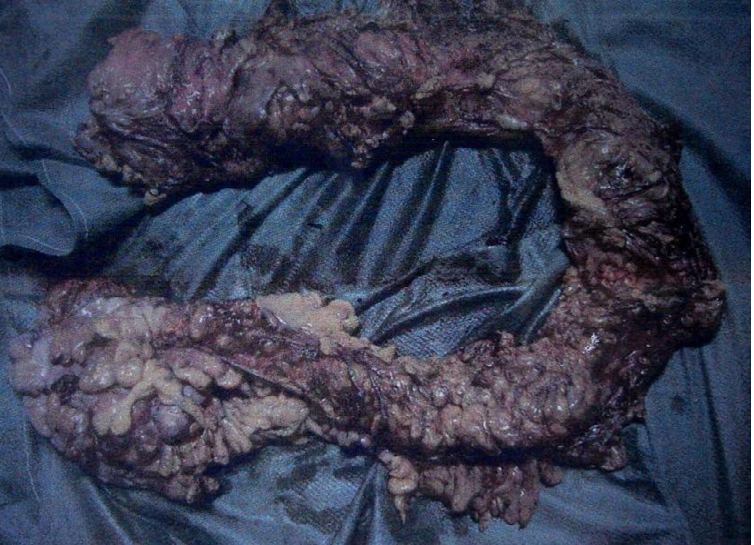
Resected Specimen with opened up distal end showing fat wrapping, strictures, and thickened wall.

Colonoscopic biopsy showed non specific inflammatory changes, AFB stain was negative. With the provisional diagnosis of tubercular stricture of colon or inflammatory bowel disease, patient was taken up for surgery. Per operatively, whole of the colon was thickened with multiple concentric strictures involving transverse colon, descending colon & recto sigmoid junction. Fat wrapping of colon was present and there were multiple small firm lymph nodes in mesentery (Fig 3). A subtotal colectomy and ileorectal anastomosis was performed. Patient did well post operatively.

**Figure 4:**
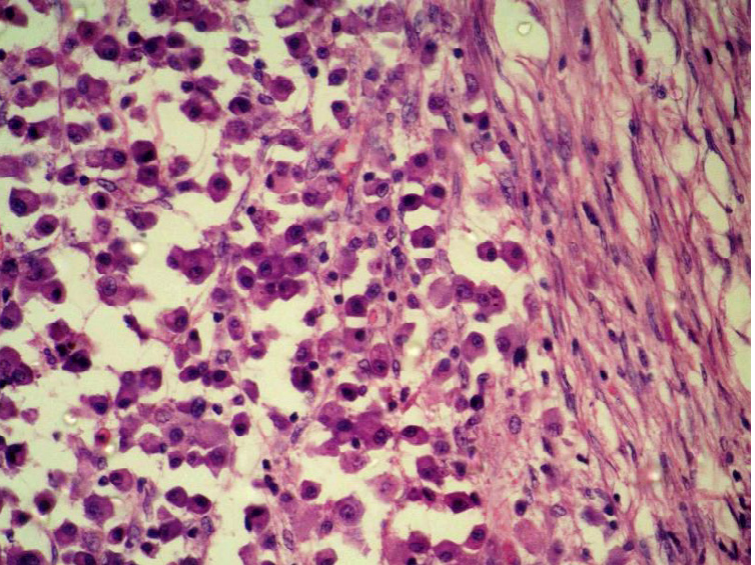
Photomicrograph showing plasma cells infiltrating colonic muscle. (HE × 200).

**Figure 5:**
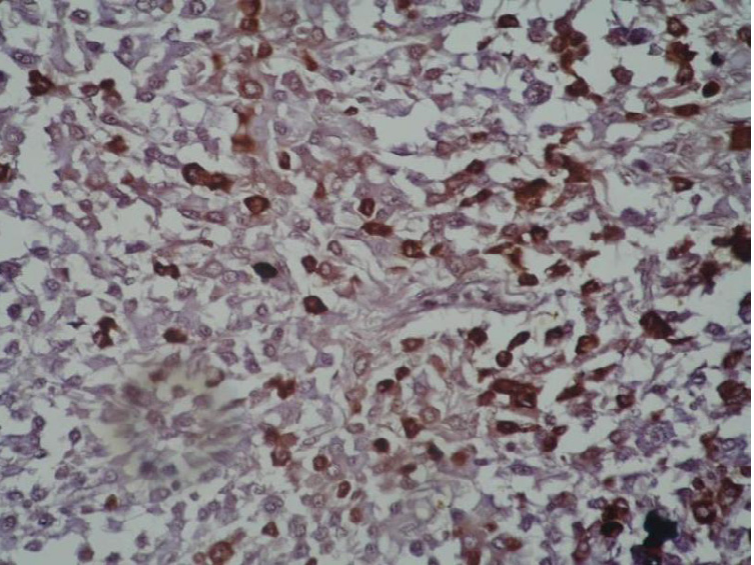
Immunostaining for kappa antibodies shows reactivity in the plasmacytoid cells (brown) (IHC × 200).

Histopathological examination of resected colon showed plasmacytoid mononuclear cells infiltrating all layers of colon along the whole length of colon especially in the strictures' areas (Fig 4). Although these cells were characterized by mild anisocytosis, their mitotic activity was very low. Overlying mucosa was ulcerated. Small intestine and colonic resected margins showed focal aggregate of plasmacytoid cells. All 3 isolated lymph nodes showed plasmacytoid cells in the sinuses and follicles in addition to a reactive lymphoid hyperplasia. Immunohistochemical staining for most of the markers (CK, S-100, Vimentin, Desmin, LCA, CD 68, CD 3, CD 20 and Chromogranin) were negative. However, immunoperoxidase staining for k chains was positive. It was negative for lambda chains (Fig 5). Based on pathological examination a diagnosis of plasmacytoma of colon was made. To exclude associated multiple myeloma, patient underwent bone marrow biopsy, bone survey and his peripheral smear, urine for Bence Jones protein and serum electrophoresis were examined and were found to be normal.

Post operatively adjuvant chemotherapy (5 cycles of melphalan 12 mg/d D1–4, and prednisolone 90 mg/d, D1–4) was given. Patient has been on regular follow up in last 17 months. He had passage of blood mixed stools 4 months back, sigmoidoscopy showed anastomotic ulceration and biopsy from ulcer showed non specific changes. Patient received conservative treatment which resulted in the resolution of symptoms. A repeat bone marrow biopsy was done 3 months back and was normal.

## Discussion

Extramedullary plasmacytoma are rare tumors. They may be solitary or may occur in association with multiple myeloma. In the latter case it may precede, accompany or follow the onset of multiple myeloma.

Diagnosis of solitary extramedullary plasmacytoma requires the exclusion of associated multiple myeloma as shown by negative Bence Jones proteins in urine, normal serum electrophoresis and normal bone marrow biopsy [5]. Our present case fulfills the above criteria as urine examination was negative for Bence Jones proteins; and the bone survey, bone marrow examination, and serum electrophoresis were also normal.

Histopathologically these lesions are characterized by monoclonal proliferation of plasma cells. Monoclonal nature of proliferation can be confirmed by demonstrating single type of paraprotein (kappa or lambda chains) in tumor cells by immunoperoxidase staining. The demonstration of single type of paraprotein in tumor cells is considered the conclusive evidence for making the diagnosis of plasmacytoma [6]. In the present case immunoperoxidase staining showed only the presence of kappa chains indicating monoclonal nature of cellular proliferation.

Histologically these tumors have to be differentiated from reactive plasmacytosis. Polyclonal nature of cells as shown by immunoperoxidase staining, characterizes reactive plasmacytosis [6]. Solitary extramedullary plasmacytoma most often occurs in nasopharynx and upper respiratory tract. Only 10% of extra osseous involvement occurs in gastrointestinal tract. In the gastrointestinal tract, stomach and small intestine are the most commonly involved sites [7]. Eighteen cases of extramedullary plasmacytoma of colon have been reported in literature however only few cases have been well documented (Table 1)

**Table 1:** Well documented cases of isolated primary plasmacytoma of colon*

Author/year	A/S	Symptoms	Duration	Site	Lymph node	Rx
Miller 1970	35 M	Anemia	6 m	Cecum	ND	Rt HC
Neilson et al 1972	82 F	Pain	5 yr	Sigmoid	+	Resection
Wing et al 1975	82 F	Pain	2 wks	As Colon	-	Rt HC
Shaw et al 1976	47 F	Diarrhea	2 wks	Cecum	+	Resection
Staples et al 1977	61M	Incidental operative finding	-	Sigmoid	-	resection
Adekunle 1978	35 M	Pain	1 yr	Cecum	+	Rt HC
Sidani et al 1985	52 M	Pain	2 m	Sigmoid	-	Resection
Present case	42 M	Diarrhea	8 m	Diffuse colon	+	STC

*Only with clinical and other details have been included.

Rt HC: Right Hemicolectomy

STC: Sub Total Colectomy

ND: Not Described

Males are more commonly affected than females as seen in other extramedullary plasmacytomas. Patients in the age group of 35 to 85 years are usually affected. Clinical presentation is variable, however most common symptom is abdominal pain. Some degree of rectal bleeding is also present. Duration of symptoms varied from a few days to as long as 10 yrs [8]. In the present case patient presented to us with an eight months history although he had similar symptoms 3 times in last 25 years. Whether these are interrelated and disease gradually progressed during this long period in not clear.

Most common findings on barium enema examination include polypoid mass or constricting lesion with or without mucosal or submucosal infiltration. Other less common findings include superficial ulcers, polyps (single or multiple), or intussusception [9,10]. In the present case whole extent of colon was involved by the tumor more so in the strictured areas. Extensive involvement of colon with multiple strictures as seen in the present case has not been reported earlier. These colonic lesions may simulate adenocarcinoma and plasmacytoma can not be diagnosed solely on the basis of radiographic findings. It may also mimic inflammatory bowel disease. However plasmacytoma may coexist with Crohn's disease [11].

Colonoscopy has not been well described in these cases. Only in one case [12] it was performed and showed polyp in sigmoid. In the present case, a non passable stricture was found 18 cm from anal verge. Biopsy taken from stricture site did not reveal any specific pathology and was not helpful.

Because of very few numbers of primary gastrointestinal plasmacytoma, at present there is no well defined treatment guideline. Localised plasmacytoma of gastrointestinal tract usually have been treated by surgical resection. As these tumors are radiosensitive, radiotherapy has also been used instead of surgery for rectal tumors with good response [13]. Chemotherapy has been used in cases of associated systemic disease. Allison et al [14] had proposed treatment of gastrointestinal plasmacytoma according to staging.

### Group A

Stage I Localized disease Resection

Stage II Lymph nodes +, residual disease Resection + radiotherapy

Stage III Distant metastases Resection+ radiotherapy+ chemotherapy

### Group B

Synchronous disease Resection if indicated, radiotherapy, and chemotherapy

### Group C

Metachronous generalized disease Chemotherapy

Long term follow up is very important as systemic disease may present later on. In the largest series of extramedullary plasmacytoma of 22 patients, 32% developed multiple myeloma after a median follow up of 1.8 years [2]. In this series 2 patients had colonic plasmacytoma and 1 patient developed multiple myeloma after 7 months.

Prognosis of these solitary extramedullary plasmacytoma tumors is generally good. In 22 pateints from MD Anderson Cancer Centre, the median survival was 9.5 years and 56% patients were free from systemic disease at 5 years [2].

As the total number of solitary extramedullary plasmacytoma of colon is very small, natural history, treatment guidelines, prognosis is not very well defined. Information regarding these cases is based on extramedullary plasmacytomas of other sites mainly nasopharyngeal and upper respiratory tract tumors.

Although these cases are rare, treating physician as well as radiologist, and pathologist should be aware of this entity as these are known to occur in non osseous sites and isolated colonic lesion may mimic carcinoma or even tubercular stricture.

## Conclusion

Plasmacytoma is known to occur in extra osseous sites. Primary colonic plasmacytoma, however, is a rare clinical entity. Primary colonic plasmacytoma presenting with multiple colonic strictures may mimic colonic tuberculosis or inflammatory bowel disease. Although these cases are rare, treating physician as well as radiologist, and pathologist should be aware of this entity

## Competing interests

The author(s) declare that they have no competing interests.

## Authors' contributions

All the authors have contributed equally.
